# Un nodule plantaire

**DOI:** 10.11604/pamj.2017.27.152.13019

**Published:** 2017-06-30

**Authors:** Amina Kissou, Badr Eddine Hassam

**Affiliations:** 1 Service de Dermatologie, Centre Hospitalier, Universitaire IBN, Sina, Rabat, Maroc

**Keywords:** Histiocytofibrome, nodule, plante du pied, Histyocytofibroma, nodule, sole

## Image en médecine

Un patient âgé de 30 ans, sans antécédents notables, s'est présenté en consultation de dermatologie pour l'apparition d'une nodule plantaire qui évoluait depuis 2 ans. L'examen clinique trouvait un nodule douloureux au niveau de la plante du pied droit. La lésion mesurait 2.5cm de grand axe, bien enchâssée dans le derme, à surface kératosique et de consistance dure (A). Il n'y avait pas d'adénopathies. Une biopsie cutanée a été faite. L'histologie avait objectivé une prolifération tumorale fuso-cellulaire, sous épidermiques, faites de faisceaux entrecroisés storiformes. Les cellules tumorales étaient allongées au cytoplasme réduit éosinophile, munies de noyaux allongés à chromatine fine avec des figures de mitoses estimée à 6 mitoses/10 champs faisant évoquer un sarcome de Darier et Ferrand (B). L'immuno-histochimie avait objectivé un marquage CD68 positif, par contre le CD34 était négatif (C,D). Le diagnostic d'une histiocytofibrome cellulaire bénin a été retenu. Le patient a bénéficié d'une exérèse totale sans récidive avec un recul de 3 ans. Dans notre cas, l'immuno-histochimie a permis de redresser le diagnostic d'une histioctofibrome bénin, dans sa forme atypique, pris initialement pour une tumeur maligne.

**Figure 1:**
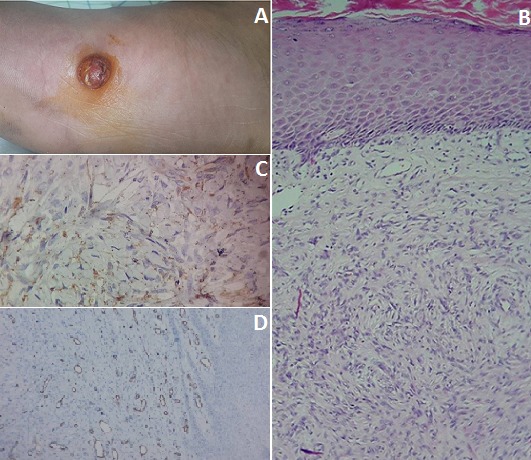
(A) nodule au niveau de la plante du pied droit; (B) une prolifération tumorale fuso-cellulaire, sous épidermiques, faites de faisceaux entrecroisés storiformes; (C) CD68 positif; (D) CD34 négatif

